# A simplified method for power-law modelling of metabolic pathways from time-course data and steady-state flux profiles

**DOI:** 10.1186/1742-4682-3-24

**Published:** 2006-07-17

**Authors:** Tomoya Kitayama, Ayako Kinoshita, Masahiro Sugimoto, Yoichi Nakayama, Masaru Tomita

**Affiliations:** 1 Institute of Advanced Bioscience, Keio University, Fujisawa, 252-8520, Japan; 2 Department of Bioinformatics, Mitsubishi Space Software Co. Ltd., Amagasaki, Hyogo, 661-0001, Japan; 3 Network Biology Research Centre, Articell Systems Corporation, Keio Fujisawa Innovation Village, 4489 Endo, Fujisawa, 252-0816, Japan

## Abstract

**Background:**

In order to improve understanding of metabolic systems there have been attempts to construct S-system models from time courses. Conventionally, non-linear curve-fitting algorithms have been used for modelling, because of the non-linear properties of parameter estimation from time series. However, the huge iterative calculations required have hindered the development of large-scale metabolic pathway models. To solve this problem we propose a novel method involving power-law modelling of metabolic pathways from the Jacobian of the targeted system and the steady-state flux profiles by linearization of S-systems.

**Results:**

The results of two case studies modelling a straight and a branched pathway, respectively, showed that our method reduced the number of unknown parameters needing to be estimated. The time-courses simulated by conventional kinetic models and those described by our method behaved similarly under a wide range of perturbations of metabolite concentrations.

**Conclusion:**

The proposed method reduces calculation complexity and facilitates the construction of large-scale S-system models of metabolic pathways, realizing a practical application of reverse engineering of dynamic simulation models from the Jacobian of the targeted system and steady-state flux profiles.

## Background

Systematic modelling has emerged as a powerful tool for understanding the mathematical properties of metabolic systems. The rapid development of metabolic measurement techniques has driven advances in modelling, especially using data on the effects of perturbations of metabolite concentrations, which contain valuable information about metabolic pathway structure and regulation [1]. A power-law approximation for representing enzyme-catalyzed reactions, known as Biochemical Systems Theory, is an effective approach for understanding metabolic systems [2,3]. Generalized Mass Action (GMA) and S-systems [4,5], which are often used as power-law modelling approaches, have wide representational spaces that permit adequate expression of enzyme kinetics [6] in spite of their simple fixed forms. Moreover since S-system forms have a smaller number of parameters than GMA forms, the S-system is an appropriate modelling framework. The derivation of an S-system model from given experimental data is a powerful tool not only for understanding non-linear properties but also for determining the regulatory structure of the system [7,8].

S-system modelling from time-course data is often difficult due to its non-linear properties. Non-linear-fitting algorithms, such as genetic algorithms or artificial neural networks, have been used to resolve this problem [9-14]. Although these methods can be applied to metabolic pathways, massive computing power is required in the case of targeted models involving a number of closely-connected, underspecified parameters [13]. Moreover, the wider the range of targeted metabolic pathways, the more likely is the occurrence of local minima due to the expansion of the parameter search space. The network-structures-segmentation method can reduce the total parameter search range in genetic network modelling [15] but it is difficult to apply this to metabolic pathways because of the close relationships between reversible reactions and because of allosteric regulation. Diaz-Sierra and Fairén have proposed an approach, based on the steady-state assumption, that allows the construction of S-system models from a Jacobian matrix of the system [16]. Since the Jacobian constrains the search range of underspecified parameters at the optimization stage, these authors' method allows efficient parameter estimation. However, the problem of an excess number of parameters requiring estimation remains unsolved.

We present an approach to power-law modelling of metabolic pathways from the Jacobian of the targeted system and steady-state flux profiles with linearization of the S-system. This reduces the number of underspecified parameters. Two numerical experiments show that the S-system model generated by this method describes similar dynamic behaviour to that indicated by conventional kinetic models.

## Methods

### Retrieving the Jacobian from time-course data

As a first step, the Jacobian must be obtained from metabolic time-course data. In this section, we summarize the method of Sorribas *et al *[17], which we use in this work.

In biochemical systems, the Jacobian can be defined as:



where **J **is the Jacobian matrix, and δ**X **represents a small perturbation and contains the concentration X_i _as its elements. The elements of the Jacobian can be obtained from perturbed time-courses using linear least-squares fitting [17,18]. This method is based on the fact that transients yield linear responses to small perturbations under steady-state conditions [5]. The mathematical basis for this is that the linear representation constitutes the first-order term of a Taylor series expansion, which is sufficiently accurate in this situation.

### Determination of the kinetic orders of the S-system

The S-system is a power-law representation constructed of two terms: the production rate and the degradation rate:



where α_i _and β_i _are rate constants, g_ij _and h_ij _are kinetic orders, and x_i _represents the concentration of a compound.

In steady state conditions, it can be expressed simply as:

*Flux *= * V*^+ ^= * V *^- ^    (3)

where Flux is the sum of the steady-state fluxes into x_i_, and V^+ ^and V^- ^are production and consumption terms, respectively.

In steady state conditions, the production term and the consumption term in Eq.(2) can therefore be represented as:



where x_j,0 _represents the steady-state concentration of x_j_.

Consequently α_i _and β_i _yield:





Defining X_i _as:

 = *y*_*i *_(*x*_1_, ..., *x*_*n*_)     (*i *= 1, ..., *n*)     (7)

the Jacobian of Eq. (2) can be represented as:



In steady state conditions, Eq. (8) can be simplified by substitution of Eq. (5) with α_i _and Eq. (6) with β_i_, giving:



and thus:



Once Jacobian *J*_ij_, x_j,0_, and Flux are given, Eq. (10) is a constraint for the determination of the kinetic orders g_ij _and h_ij_. Savageau has described the linearization of S-system as an "F-factor" for stability analysis [19]. We use this representation to estimate parameter values.

In most cases g_ij _and/or h_ij _are available from the structure of the metabolic pathway; however, in the absence of known kinetic orders, parameter estimates are needed to determine them. In such cases Eq. (10) is adopted as limiting the parameter search range.

## Results

### Case study 1: a linear biochemical pathway

**Figure 1:**
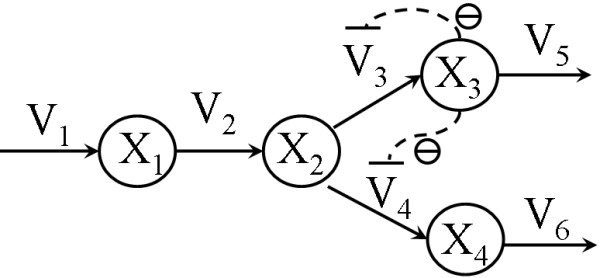
**The pathway of galactose metabolism [20]**. Substances (X_1–5_) represent the following metabolites in the paper referenced: X_1_, external galactose (GA_E_); X_2_, internal galactose (GA_I_); X_3_, galactose 1-phosphate (G1P); X_4_, UDP-glucose (U_GL_); and X_5_, UDP-galactose (U_GA_). The reactions are V_TR_, transporter of galactose; V_GK_, catalyzed by galactokinase; V_GT_, catalyzed by galactose-1-phosphate uridyltransferase; V_EP_, catalyzed by UDP-galactose 4-epimerase. X_3 _is an inhibitor whose rate is given as V_GK_. The kinetic equations, systems parameters, and initial conditions of this model are shown in Appendix 1.

In the first case study, we applied this approach to the published biochemical model of yeast galactose metabolism shown in Figure 1[20]. This model consists of five metabolites, and four enzyme reactions mainly described by Michaelis-Menten equations. The kinetic equations, systems parameters, and initial conditions are listed in Appendix 1.

In this case, all parameters of the S-system model were determined, without the need for estimation. The biochemical model could be converted into the following S-system form.









Since X_1 _is an independent variable, it was omitted from the listed rate equations.

The following constraints were provided from the pathway structure: h_22 _= g_32_, h_23 _= g_33_, h_33 _= h_43 _= g_53_, h_34 _= h_44 _= g_54_, h_54 _= g_44_, h_55 _= g_45_, β_2 _= α_3_, β_3 _= β_4 _= α_5_, and α_4 _= β_5_. The steady-state concentrations were: X_1,0 _= 0.50 mM (fixed value), X_2,0 _= 0.146 mM, X_3,0 _= 0.007 mM, X_4,0 _= 0.817 mM, and X_5,0 _= 0.243 mM. The steady-state fluxes were: V_1 _= V_2 _= V_3 _= V_4 _= 0.081 mM/min. The Jacobian was:



Our method was able to generate the S-system model of the linear pathway from the steady-state metabolite concentrations, the steady-state fluxes, and its Jacobian. For example, g_21 _and g_32 _were determined by:





Because h_22 _is equal to g_32_,





In this modelling process, all the parameters were determined by simple calculations of this kind. The resulting S-system model was:









### Case study 2: A branched biochemical pathway

**Figure 2:**
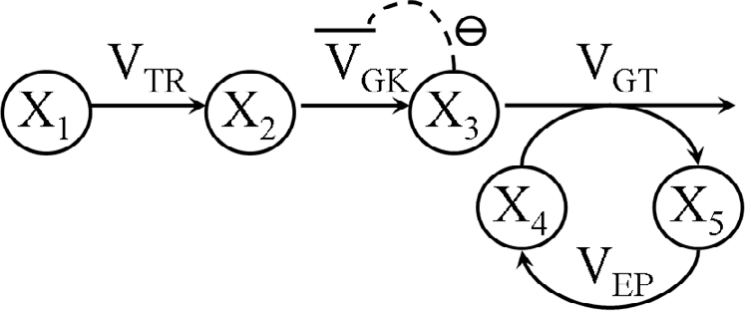
**The branched pathway**. X_3 _is an inhibitor of both V_3 _andV_4_.









The branched biochemical pathway shown in Figure 2 was tested in the second example. This pathway has the typical features of a biochemical pathway: branching, feedback regulation, and product inhibition (see Appendix 2). X_3 _is the inhibitor of both V_3 _and V_4_.

Most of the parameters were obtained by our method. However one parameter could not be determined by calculation and a parameter estimation method was used. The model could be converted into the following S-system form:

The following constraint was provided by the pathway structure: h_11 _= g_21_. Steady-state concentrations were: X_1,0 _= 0.067, X_2,0 _= 0.049, X_3,0 _= 0.081, X_4,0 _= 0.041, and steady-state fluxes were: V_1 _= V_2 _= 0.1, V_3 _= V_5 _= 0.043, and V_4 _= V_6 _= 0.057. The Jacobian was:



More than half of the parameters were obtained by simple calculations using the steady-state concentrations, the steady-state fluxes, and the Jacobian. The following four parameters could not be determined: α_3_, g_33_, β_3_, and h_33_. For example, h_11 _is a parameter which could be determined by:





Because g_33 _is a parameter that could not be determined by a calculation, an estimate provided by some constraint was needed. As α_3_, h_33_, and β_3 _could be treated as dependent parameters, once g_33 _was obtained all four parameters were determined:





Moreover, the search range of g_33 _could be limited by the definition that h_33 _has a positive value since X_3 _is the substrate of the reaction V_3_. The search range of g_33 _was set from 0 to -0.95. When the number of underspecified parameters was one whose search range was limited, a linear-fitting algorithm could be used to fit the time-course data in the original kinetic model. In this modelling, g_33 _was determined by a linear optimization method. Consequently, the following S-system model was generated:









The list of parameters used in the above calculation is shown in Table 1.

**Table 1:** The parameters of the S-system model in case study 2

Parameter	Calculated	Estimated	Determined or Estimated
α1	0.1	0.1	determined
β1,α2	0.955	0.955	determined
h11, g21	0.833	0.833	determined
β2	0.908	0.908	determined
h22	0.861	0.861	determined
h23	-0.154	-0.154	determined
α3	0.464	0.471	determined from g33
g32	0.892	0.892	determined
g33	-0.124	-0.118	estimated
β3	0.345	0.351	determined from g33
h33	0.827	0.833	determined from g33
α4	0.453	0.453	determined
g42	0.838	0.838	determined
g43	-0.178	-0.178	determined
β4	0.995	0.995	determined
h44	0.898	0.898	determined

### Comparing transient dynamic responses after perturbation

**Figure 3:**
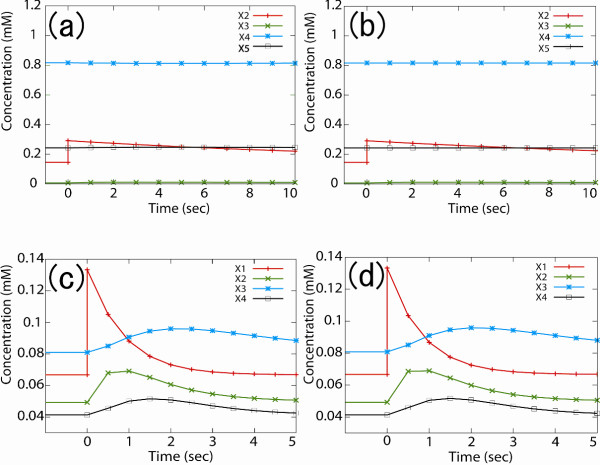
**metabolite changes in response to perturbation of one metabolite by 100%**. (a) and (b) represent the changes in metabolites in case study 1 when X_2 _is the perturbation target, derived using the reference and S-system model, respectively. (c) and (d) are the changes in case study 2 when X_1 _is the perturbation target given by the reference and S-system model, respectively.

To evaluate the versatility of this method, the transient dynamics of the S-system model in response to various perturbations were compared with those of the original Michaelis-Menten model, by calculating the mean relative errors (MRE):



where n is the number of metabolites in the model, m, the number of sampling points in the time course, x'_i_(t) represents the time course calculated from the created S-system model, and x_i_(t), the time course calculated from the original Michaelis-Menten model. In this experiment there were 10 sampling points, the interval between sampling points was 0.5, and X_2 _in case study 1 and X_1 _in case study 2 were the targets of perturbations ranging from 0% to 200%. The time-courses of metabolites in response to perturbations of 100% are shown in Figure 3. In both examples, similar dynamic behaviour was observed in the S-system model and the reference model as a response to the perturbation.

**Figure 4:**
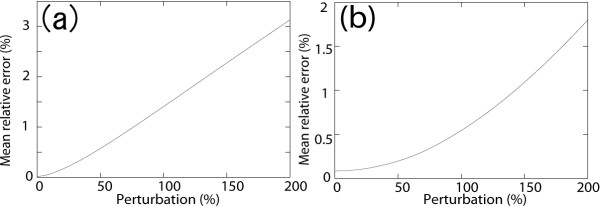
**Changes in the mean relative error (MRE)**. Perturbations of the targeted metabolite ranging from 0% to 200% were applied. (a) Evolution of MRE in case study 1, in which X_2 _was the perturbation target. (b) Evolution of MRE in case study 2, in which X_1 _was the perturbation target.

The changes of MREs in response to the perturbation range are shown in Figure 4. The initial MREs with no perturbation were within 1%. Although a slight increase in MREs was observed in both case studies, they remained within 4% at a perturbation range of 200%.

## Discussion

Power-law modelling from time-course data of metabolite concentrations often requires parameter estimates that depend on the size of the target metabolic network. Especially when developing a large-scale metabolic model, this requires problem-specific simplifications. Our method can reduce the number of underspecified parameters by using steady-state flux profiles and the Jacobian of the targeted system derived from time courses of metabolites, and is thus suitable for large-scale power-law modelling. To validate our methods we used two existing biochemical pathway models, the straight and branched pathway models, described by dynamic equations. In the S-system, modelling of a linear metabolic pathway with 12 parameters (case study 1), our method determines all parameters accurately, whereas the method developed by Diaz-Sierra and Fairén [16] leaves at least four parameters underspecified that include error correction parameters. In the case of the branched metabolic pathway with 16 parameters (case study 2), our method determines all but one parameter, whereas the method of Diaz-Sierra and Fairén has 12 underspecified parameters that include error correction parameters. The case studies demonstrate that our method of developing S-system models from time series can reduce the number of underspecified parameters more efficiently than the previously reported method [16]. Furthermore, the perturbation response experiments show that the models created by our method can reproduce dynamics similar to the reference models, since the MRE was around 3% when the perturbation range was 200% (Fig. 4). S-system models generated by our method can provide accurate simulations within a wide range of the steady-state point. This limitation does not prevent the modelling and analysis of metabolic pathways, as it does with many power-law metabolic models [4,5].

Robustness against experimental noise is an important requirement for the practical application of modelling methods. Since the Jacobian is rather sensitive to the noise in time series [18], a robust corrective response to the numerical errors contained in a Jacobian is important in developing a model involving its use. In the method of Diaz-Sierra and Fairén [16], error correction parameters are incorporated into the S-system model to reduce the effect of experimental noise. However, this approach may lead to an increase in the number of underspecified parameters. As an alternative approach to reducing the experimental noise in time-course data, the estimation of appropriate slopes using a non-linear neural network model is effective [10], and can provide an error-controlled time course that enables the Jacobian to be obtained with high accuracy. Methods for obtaining accurate estimates of the specific effects of general types of perturbation have been discussed [21] and might enable more precise analysis of data such as time-scale metabolite concentrations obtained under perturbations.

To assess the robustness of our method against experimental noise, we measured the calculation error when numerical errors were manually inserted into the Jacobian or steady state fluxes. Table 2 summarizes the MREs of the simulated time trajectories of the S-system and the original Michaelis-Menten model. It is evident that the Jacobian is quite sensitive to numerical error because of the direct effect on a kinetic order of a Jacobian. Furthermore, the steady-state flux profile data may include experimental noise. However, the models created by our method are relatively robust to errors in the Jacobian and steady-state fluxes, indicating that these methods will be useful in practice.

**Table 2:** Mean relative errors (MREs) as a function of experimental errors. The MREs of the S-system models and the original models were measured in case study 2. The time-course data obtained from the models included the perturbation-of-state variable in order to examine the difference in dynamic response between the S-system model and the original Michaelis-Menten model. The Jacobian and steady-state fluxes were reproduced with a 100% numerical error. Numerical errors for the Jacobian were inserted equally into all the elements of the Jacobian. X_1_**was the target of the perturbation. The time-course data were obtained from the S-system model where X**_1_was perturbed by an increase of 50%. Ten time points were sampled for the calculation, with an interval of 0.5 s between them. The MRE was calculated from the time-course data in the S-system model and the original Michaelis-Menten model. In the case of the branched biochemical pathway (case study 2), the Jacobian was increased by 100%, and the three steady-state fluxes, J_1–2_, J_3–5_, and J_4–6_represent the flux through V_1 _and V_2_, the flux through V_3 _and V_5 _and the flux through V _4 _and V_6_, respectively.

Experimental Data	Size of Error (%)	MRE (%)
Jacobian	100	5.06
J_1–2_	100	0.55
J_3–5_	100	1.86
J_4–6_	100	1.65

Sorribas and Cascante proposed a method for identifying regulatory patterns using a given set of logarithmic gain measurements [7]. In their paper, they suggested that one strategy for selecting possible patterns is to perform perturbation experiments and to measure the corresponding dynamic response. For practical application of this approach, it is crucial to develop appropriate ways of performing the required experiments; however, measuring the logarithmic gain resulting from various steady-state fluxes is not practical due to a lack of exhaustive measurement method. Our approach can be used to develop reasonably accurate models of metabolic pathways by using a single set of appropriate steady-state flux profiles. Although steady-state flux profile data remains difficult to be measured directly and comprehensively, several methods of measuring steady-state flux profiles by using isotopes have been developed [22,23]. Our method assumes that the Jacobian obtained is accurate. Therefore it is important to obtain time-course data reflecting transient dynamics after a suitably small perturbation in which the Jacobian behaves in a linear manner [17].

Comprehensive metabolome data will undoubtedly accumulate as a consequence of advances in metabolic measurement techniques [24-26]. In our laboratory we have developed a high-throughput technique using capillary-electrophoresis mass spectrometry that provides effective time-course data involving a few hundred ionic metabolites [27,28]. Our method promises to provide high-throughput modelling of large-scale metabolic pathways by exploiting the accumulating metabolome and steady-state flux profile data along with the anticipated developments in metabolome measurement techniques.

## Conclusion

Our method provides stable and high-throughput S-system modelling of metabolic pathways because it drastically reduces underspecified parameters by employing the steady-state flux profile and Jacobian retrieved from time-course data. S-system models generated by this method can provide accurate simulations within a wide range around the steady-state point. In combination with the metabolome measurement techniques it should permit high-throughput modelling of large-scale metabolic pathways.

## Appendices

### Appendix 1: Biochemical model of yeast galactose metabolism

The model of the yeast galactose utilization pathway was constructed by Atauri *et al*. [20], whose reaction map is presented in Figure 1.

The rate equations of the model are:









where the rate expressions are:









The parameters used are available in reference [20]. The calculated steady-state conditions were: X_1_; 0.5 mM (fixed), X_2_; 0.146 mM, X_3_; 0.00703 mM, X_4_; 0.817 mM, X_5_; 0.243 mM, and the flux through the pathway; 0.0081 mM·s^-1^.

### Appendix 2: Branched biochemical pathway

The rate equations of the branched model the reaction scheme of which is shown in Figure 2 are:

 = *V*_1 _- *V*_2_

 = *V*_2 _- *V*_3 _- *V*_4_

 = *V*_3 _- *V*_5_

 = *V*_4 _- *V*_6_

where the rate expressions are:

*V *= 0.1











## Competing interests

The author(s) declare that they have no competing interests.

## Authors' contributions

Kitayama contributed to the development of the modelling method. Kinoshita supported the development of the mathematical theory of this method and wrote this manuscript. Sugimoto designed two experiments for method verification. Nakayama provided the basic ideas and directed the project, and Tomita was the project leader.
